# Effects of light on human circadian rhythms, sleep and mood

**DOI:** 10.1007/s11818-019-00215-x

**Published:** 2019-08-20

**Authors:** Christine Blume, Corrado Garbazza, Manuel Spitschan

**Affiliations:** 1 Centre for Chronobiology, Psychiatric Hospital of the University of Basel (UPK), Basel, Switzerland; 2 Transfaculty Research Platform Molecular and Cognitive Neurosciences (MCN), University of Basel, Basel, Switzerland; 3 Centre for Cognitive Neuroscience, University of Salzburg, Salzburg, Austria; 4 Department of Experimental Psychology, University of Oxford, Oxford, UK

**Keywords:** Circadian rhythms, Natural light, Artificial light, Depression, Light therapy, Zirkadiane Rhythmen, Natürliches Licht, Künstliches Licht, Depression, Lichttherapie

## Abstract

Humans live in a 24-hour environment, in which light and darkness follow a diurnal pattern. Our circadian pacemaker, the suprachiasmatic nuclei (SCN) in the hypothalamus, is entrained to the 24-hour solar day via a pathway from the retina and synchronises our internal biological rhythms. Rhythmic variations in ambient illumination impact behaviours such as rest during sleep and activity during wakefulness as well as their underlying biological processes. Rather recently, the availability of artificial light has substantially changed the light environment, especially during evening and night hours. This may increase the risk of developing circadian rhythm sleep–wake disorders (CRSWD), which are often caused by a misalignment of endogenous circadian rhythms and external light–dark cycles. While the exact relationship between the availability of artificial light and CRSWD remains to be established, nocturnal light has been shown to alter circadian rhythms and sleep in humans. On the other hand, light can also be used as an effective and noninvasive therapeutic option with little to no side effects, to improve sleep, mood and general well-being. This article reviews our current state of knowledge regarding the effects of light on circadian rhythms, sleep, and mood.

## Zusammenfassung

Der Mensch lebt in einer 24-Stunden-Umgebung, in der sich Licht und Dunkelheit abwechseln. Unser zirkadianer Schrittmacher in den suprachiasmatischen Nuclei (SCN) des Hypothalamus synchronisiert unsere inneren biologischen Rhythmen mit dem Tagesverlauf des Sonnenlichts über Leitungsbahnen, die von der Netzhaut zu den SCN ziehen. Rhythmische Schwankungen in der Umgebungsbeleuchtung beeinflussen unser Verhalten, etwa den Wechsel zwischen Ruhe im Schlaf und Aktivität im Wachzustand, und dessen zugrunde liegenden biologischen Prozesse. In jüngster Zeit hat die Verfügbarkeit von künstlichem Licht die Lichtumgebung erheblich verändert, insbesondere während der Abend- und Nachtstunden. Dies kann das Risiko für die Entstehung von circadianen Schlaf-Wach-Rhythmusstörungen („circadian rhythm sleep-wake disorders“, CRSWD) erhöhen, die oft durch eine Fehlausrichtung bzw. einen Versatz zwischen endogenen tageszeitlichen Rhythmen und dem externen Hell-Dunkel-Zyklus verursacht werden. Zwar ist der genaue Zusammenhang zwischen der Verfügbarkeit von künstlichem Licht und CRSWD noch nicht geklärt, doch es hat sich gezeigt, dass nächtliches Licht den zirkadianen Rhythmus und den Schlaf beim Menschen verändert. Andererseits kann Licht auch als effektive, nichtinvasive Therapieoption mit geringen bis keinen Nebenwirkungen eingesetzt werden, um Schlaf, Stimmung und Allgemeinbefinden zu verbessern. Der vorliegende Beitrag gibt einen Überblick über den aktuellen Wissensstand hinsichtlich der Auswirkungen von Licht auf zirkadiane Rhythmen, Schlaf und Stimmung.

## Anatomical architecture of the circadian system

The central master-clock in mammalian species, including humans, is the suprachiasmatic nuclei (SCN), a paired structure in the hypothalamus with a volume just about 0.25 mm^3^ per nucleus (e.g. [45, 57, 84]). Within the mammalian SCN, a molecular oscillator keeps the clock oscillating at its normal pace. The basis of this oscillator is two interconnected molecular feedback loops of clock gene expression, a detailed description of which is beyond the scope of this review though (see [12] for a detailed explanation).

**Fig. 1:**
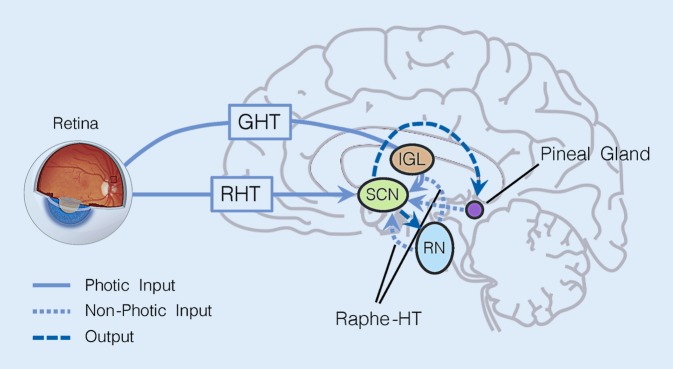
Input and output pathways to/from the suprachiasmatic nuclei (*SCN*). The photic input pathways that relay information about the intensity and spectral composition of ambient light are the retinohypothalamic tract (*RHT*) and the geniculohypothalamic tract (*GHT*), which connects retina and SCN via the intergeniculate leaflet (*IGL*) in the thalamus. Additionally, the SCN also receive non-photic information from the raphe nuclei (*RN*) via the raphe-hypothalamic tract (*raphe-HT*) and from the pineal gland. The main output is from the SCN to the serotonergic raphe nuclei (*RN*, receive information about the phase of the circadian clock and regulate vigilance state of the body) and the pineal gland, where melatonin is produced. Input and output pathways form reciprocal loops

Successful interaction between body and environment however needs more than just a central clock; it also requires input pathways relaying information about the environment and the body to the SCN to achieve adequate entrainment as well as output pathways communicating timing information to the body to synchronise bodily processes with the circadian phase (Fig. 1).

The most important zeitgeber (from German, something that “gives time”) reaching the SCN is ambient light in the environment. In addition to processing visual stimuli in the environment, allowing us to see, the retina carries this photic information via the retinohypothalamic tract (RHT) to the SCN. The SCN also receive non-photic information from within the body. Here, the involved pathways comprise the geniculohypothalamic tract (GHT), which communicates both non-photic and photic information (via the intergeniculate leaflet; IGL), and the raphe-hypothalamic tract (raphe-HT). Additionally, SCN activity is also modulated by non-photic information via neurotransmitters and hormones such as serotonin [54] and melatonin [23], and from peripheral clocks in other tissues (see [55] for an overview).

SCN neurons adjust their circadian phase (of neural activity) according to the input of ambient light levels and its spectral composition and communicate this information via humoral and autonomic nervous system signals to the rest of the body. These output pathways are also reciprocal and thus feed information back to the SCN: The SCN-serotonin-producing raphe nuclei(RN)-SCN loop as well as the SCN-melatonin-producing pineal gland-SCN loop (Fig. 1). More specifically, the RN can alter vigilance levels in accordance with circadian phase via serotonergic wakefulness-promoting projections to the hypothalamus and the cortex [30, 56].

The SCN also projects to the pineal gland, where the sleep-facilitating hormone melatonin is produced during the biological night, thereby modulating the diurnal variations between wakefulness and sleep [23]. In addition to the pathway between retina and SCN, there is recent evidence from animal studies showing that also the habenula in the thalamus is innervated by retinal projections [38, 110] which may specifically mediate mood-related non-visual effects of light.

## Fundamentals of light

To understand the effects of light on the human physiology, it is important to understand light. Briefly, light is radiation in a specific range of the electromagnetic spectrum. It is best and most completely described by its spectral distribution, which quantifies the amount of energy (or the number of photons) as a function of wavelength (with visible light in the wavelength range between 380 and 780 nm).

**Fig. 2:**
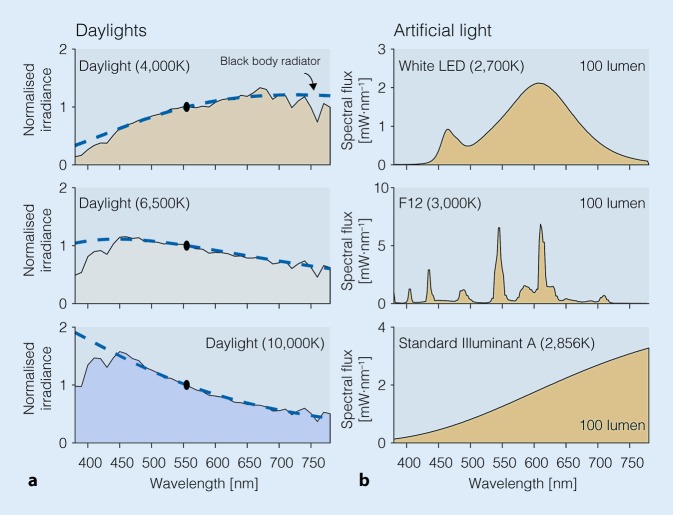
Spectral power distributions of common light sources in our environment. **a** Spectral power distributions of daylights at different correlated colour temperatures (CCT; 4000 K; 6500 K; 10,000 K). Spectra are normalised to 555 nm. **b** Spectral power distributions of a white LED (*top*), a fluorescent source at 3000 K (*middle*), and an incandescent source (tungsten-filament; 2856 K, *bottom*). All three artificial sources have the same luminous flux (normalised to 100 lm), and approximately the same colour temperature (2700–3000 K), but the spectra are very different in shape and scale (see *y* *axis*)

During the day, light intensities outside can reach illuminances up to 100,000 lx in direct sunlight and 25,000 lx in full daylight. Light intensities in closed rooms are considerably lower and standard office lighting is only ~500 lx, often lower [37, 81]. The spectrum of daylight, which is light from the sun filtered by the atmosphere is relatively broadband in its distribution (Fig. 2a). The availability of daylight depends on geographical location and season. In the timeframe of human evolution, it is a rather recent development that light can be available during all times of day through artificial light. Artificial light allows for illuminating indoor and outdoor spaces. It comes in many forms, e.g. incandescent, fluorescent, or light-emitting diode (LED) lighting. While light generated by these technologies may all appear “white”, the underlying spectra are rather different (Fig. 2b). The reason why many different types of spectra might have the same appearance lies in the retina. Critically, different spectra, even if they create the same visual impression, may vary in their chronobiological effects on the circadian clock.

It is important to keep in mind that there are multiple ways how light is quantified and reported in the literature in particular when focussing on its repercussions on human physiology. For example, while the absolute spectral distribution of a light is the most complete description, many investigators report the illuminance (in lux [lx]), or the correlated colour temperature, which is the temperature of a hypothetical black-body radiator with the same colour as the light source in question. Unfortunately, until recently, there have been no standard quantities that experimenters were asked to report, and therefore, summarising the chronobiological and somnological literature on the effects of light remains a challenge. Recently, the *Commission International de l’Eclairage *(CIE), the international standard body for quantities related to light, issued a new standard containing a reference framework for quantifying the effects of light on non-visual functions [31]. In practice, experimenters employing light as an intervention should report, at a minimum, the spectral power distribution of the light, as seen from the participant’s point of view. Detailed minimum guidelines are given in [83]. 

## Photoreceptors in the retina

**Fig. 3:**
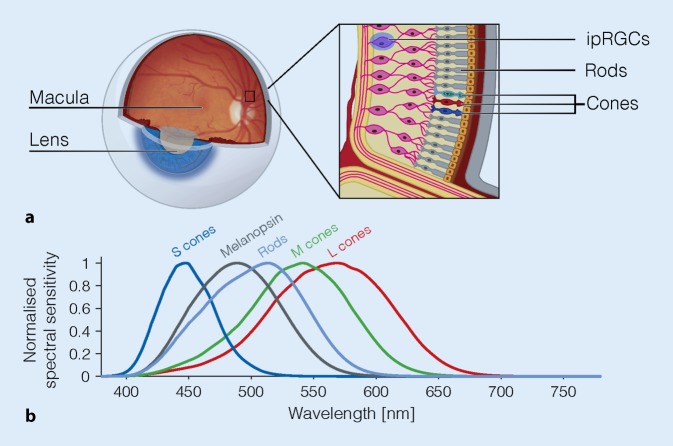
Overview of the retina photoreceptors. **a** Schematic view of the eye with the retina at the back of the eye (the fundus), containing cones, rods and the intrinsically photosensitive retinal ganglion cells (*ipRGCs*) expressing the photopigment melanopsin. **b** Spectral sensitivities of the photoreceptors in the human eye

In humans, the known effects of light on circadian rhythms and sleep are all, without exception, mediated by the retina. The retina is a fine layer of nerve tissue at the back of our eyes, containing specialised photoreceptors (Fig. 3a). The so-called cones exist in the highest density in the centre of the retina—the fovea. There are three types of cones, differing in their preference for light at specific wavelengths (Fig. 3b): The long-wavelength-sensitive cones (L cones), the medium-wavelength-sensitive cone (M cones) and the short-wavelength-sensitive cones (S cones). Cones allow us to see colour, spatial detail and motion at light levels typical for daytime. Rods, by contrast, are suppressed at daytime light levels and only signal at light levels typical for twilight and darker. Rods are absent in the fovea, cannot distinguish between different colours and only allow for rudimentary vision.

Cones and rods are not the only photoreceptors in the retina. A small fraction of secondary neurons in the retina—the retinal ganglion cells (RGCs), which integrate information and send it to the brain via the optic nerve—express the photopigment *melanopsin *[62]. Melanopsin is a short-wavelength-sensitive pigment with a peak spectral sensitivity near around 480 nm [4], rendering some RGCs intrinsically photosensitive [79]. These *i*ntrinsically *p*hotosensitive retinal ganglion cells (ipRGCs) are thought to mediate most effects of light on the circadian clock. However, ipRGCs are not independent of rod and cone input. Rather, they also receive information from these receptors, suggesting that ipRGCs indeed act as “integrators of information” regarding the light environment across a wide range of wavelengths and light levels. Surprisingly, the input from the S cones into the ipRGCs has a negative sign [32]. In humans, this has the paradoxical consequence that increases in S cone activation lead to a dilation of the pupil [80, 100], which is also controlled by the ipRGCs.

It has long been thought that cones and rods mediate what is typically considered “vision” (seeing colour, motion, spatial detail), and that melanopsin mediates the “other”, non-visual effects of light, i.e. melatonin suppression, circadian phase shifting, and alertness. However, at second sight, this dichotomy breaks down. There is now converging evidence that melanopsin signals reach the primary visual cortex (V1) [82], where they may contribute to and modulate our visual perception [20, 25, 109].

It is important to keep in mind that the retinal photoreceptors experience an altered version of the light relative to the cornea, the front surface of the eye. This is because the eye itself contains filters. In the centre of the retina, this includes the macular pigment, which is present in the fovea but drops off in the peripheral retina. More importantly for ipRGCs, the crystalline lens and ocular media filter out short-wavelength light. This natural “blue-blocking” filter increases density with increasing age, with less and less short-wavelength light reaching the retina.

While the field of vision science has a long history (>150 years) in examining how different types of light stimuli are encoded, processed and perceived, we still remain largely in the dark about many aspects of the effects of light on the circadian clock. The discovery that the production of melatonin is suppressed in humans in response to light dates back to only 1980 [51]. Teasing apart how the different elements in the retina contribute to the effects of light on circadian rhythms, sleep and mood remains an important challenge.

## Effects of light on the circadian clock

Two effects of light have been interrogated extensively in human circadian and sleep research: (1) the acute suppression of melatonin in response to light exposure and (2) the ability of light exposure to shift circadian phase. However, these two effects are not arising from a unitary pathway resulting in a direct relationship between melatonin suppression and phase shifts. There is now accruing evidence that they may be indeed separable [63]. As a consequence, one should not be used as a proxy for the other [106].

The system mediating melatonin suppression has a spectral sensitivity that is broadly consistent with the spectral sensitivity of melanopsin [17, 60, 88]. Similarly, the spectral sensitivity of circadian phase shifting shows its maximal effect near the peak spectral sensitivity of melanopsin [101]. However, this does not imply that cones and rods may not participate in these non-visual effects of light. Indeed, there is evidence that cones do contribute, though at a different time scale than the ipRGCs [42].

The effects of light on the phase of the circadian clock depend on the timing of light exposure. This is formally summarised in the phase response curve (PRC), which describes the amount of phase shift (in minutes and hours) achieved by exposure of light at a given circadian phase. Roughly speaking, the effect of morning light is that it advances the clock, while evening and night light delays the clock. The human circadian system integrates across multiple light exposures as short as five minutes [48], even intermittent bright light exposure can shift the circadian phase [43, 66]. It has been shown that under certain circumstances, a train of very brief flashes light flashes on the millisecond scale can cause circadian phase shifts which are larger than those caused by continuous light [59, 108].

Both melatonin suppression and circadian phase shifts are modulated by the “photic history”, i.e. the amount of light seen during the day [27, 44, 77]. The long-term adaptive influences of the “spectral diet” in the real world remain an important area of investigation [93].

## Effects of light on sleep

The human sleep–wake cycle, that is periods of sleep during the night and wakefulness during the day, is one of the most prominent examples of a circadian behavioural pattern. It results from the interaction between two factors: the circadian drive for wakefulness and the homeostatic sleep pressure. The interaction between this circadian “process C” and the homeostatic “process S” has been conceptualised in the widely known “two-process model of sleep” [13, 15], which accounts for the timing and intensity of sleep in many experimental settings. Indeed, in well-controlled studies the circadian pacemaker in the SCN and the sleep homeostat have been shown to interact in a fashion designed to allow for consolidated periods of wakefulness and sleep during day and night, respectively (e.g. [35]). Specifically, the activity of the circadian pacemaker is aligned to counteract the increasing sleep pressure resulting from sustained wakefulness during daytime. Likewise, the nocturnal increase in circadian sleep tendency counteracts the decrease in sleep propensity resulting from accumulated sleep thereby supporting a consolidated phase of nocturnal sleep.

As outlined above, light is the key zeitgeber in the circadian system and interacts with the master clock in the SCN via non-image-forming pathways connecting retina and SCN. Unsurprisingly, light therefore also affects sleep. Natural daylight at high intensities as experienced outside buildings has previously been shown to (1) advance the timing of sleep to earlier hours, (2) affect the duration of sleep, and (3) improve sleep quality. More precisely, the phase-advancing effects of daylight have for example been reported by Roenneberg and colleagues [67] who, using questionnaire data, found that each additional hour spent outdoors advanced sleep by ~30 min. Despite light being the strongest zeitgeber, this phase-advance could also result from physical exercise during daytime [102, 105], which is often confounded with time spent outdoors. The relative contributions of light and physical activity remain to be determined. Moreover, light exposure during the day has also been shown to affect sleep duration. Here, shorter daylight exposure and longer nights are associated with a longer biological night as indexed by the duration of melatonin secretion, and thus longer sleep duration [85, 94, 95], which may also reflect a seasonality effect [104]. Likewise, exposure to daylight has been shown to increase sleep duration, possibly by advancing sleep timing [16]. Beyond this, sleep quality is also related to light exposure during the day. Several studies report that daytime exposure to white light enriched in short-wavelength content was associated with increased evening fatigue [91], and sleep quality [16, 39, 91], decreased sleep-onset latency [39], and increased slow-wave sleep accumulation [92], which is related to the dissipation of the homeostatic sleep pressure [1, 14, 34]. However, also the timing of light exposure seems to matter for sleep. In this context, Wams and colleagues [92] report that participants with later exposure to light >10 lx had more nocturnal awakenings and less slow-wave sleep. In sum, research seems to agree that daylight (at high intensities) is beneficial for sleep.

## Exposure to artificial lighting, smartphones and visual display units

In addition to natural daylight, humans are nowadays also exposed to a considerable amount of artificial light. This is particularly the case in the evening hours, i.e. when the circadian system is most sensitive to light-induced phase delays. Thereby, artificial light can delay the timing of the circadian clock and thus sleep [102]. Indeed, light from LED screens has repeatedly been suggested to interfere with sleep and the physiological processes involved (e.g., melatonin secretion [24]). Chang and colleagues [26] for example found that reading a book from an e‑reader for four hours before sleep increased sleep onset latency, reduced evening sleepiness, melatonin secretion as well as next-morning alertness, and delayed the timing of the biological clock, which is also in line with other findings [72, 107]. It should be noted though that exposure to the “circadian-active” light source was very long in these studies (4–6.5 h) and it is unclear whether the same results can be expected for shorter exposures.

Evaluating sleep objectively with electroencephalography (EEG), Münch and colleagues [58] found that exposure to short-wavelength light for two hours starting 3 h before habitual bedtime first lead to decreased slow wave activity (SWA) and thus shallower sleep. From this, the authors concluded that the alerting effects of short-wavelength light persist into sleep, which is in line with findings by Chellappa and colleagues [28], who reported a decrease in homeostatic sleep pressure following short-wavelength light exposure in the evening. However, short-wavelength light exposure in the evening was also associated with increased SWA later during the night, suggesting a possible compensatory mechanism [58].

Also, the effects of evening light exposure do not seem to be independent from exposure during the preceding day. More specifically, Rångtell and colleagues [64] examined the effects of reading a novel on a tablet computer (~102 ± 41 lx, 7718 K) vs. in a physical book (~67 ± 50 lx, 2674 K) for two hours following prolonged (6.5 h) exposure to bright light (~569 lx, 3149 K) between 2:30 pm and 9 pm. Contrasting other findings, the light from the tablet did not suppress melatonin or alter subjective and objective sleep parameters. Note though that also exposure was shorter than in studies that reported significant effects [26, 72, 107].

Several studies have reported that smartphone ownership and use before bedtime may be associated with more self-reported sleeping problems [74], decreased sleep efficiency, longer sleep onset latency and poor sleep quality [29], and delays sleep thereby also shortening sleep duration [29, 50, 74]. Modern smartphones contain a “night shift” feature changing the colour balance in the evening hours (Infobox 1 for details). How much of the reported detrimental effect of smartphone use on sleep is due to light per se, or to some other feature (e.g. psychological engagement), is currently not known.

### Infobox 1 Smartphones and sleep

Smartphone use may delay sleep onset. One factor is the light emitted by their screens, but another may also be its entertaining character or related psychological effects, or both. Using the “night shift” mode of modern smartphones, the colour balance of the screen can be shifted to “warmer” and orangeish colours depleted in short-wavelength light. On a recent iPhone 7, this amounts to a reduction of melanopsin activation by 67% at full display brightness. This might seem like a large reduction at first, though by simply dimming the smartphone to its minimum level, the melanopsin activation can be reduced to less than 1% of the activation at maximum display brightness. Whether or not the “night shift mode” has an appreciable effect on the circadian system and how it interacts with other properties of smartphone use is currently not known. Recent research using so-called metameric displays, which do not differ in their appearance but only differ in the amount that they stimulate melanopsin, show that the non-visual properties of light can be modulated independently of visual appearance [3, 78].

## Effects of light on mood

Mood variations have been shown to be influenced by a complex and non-additive interaction between circadian phase and the duration of prior wakefulness. Specifically, relatively moderate changes in the timing of the sleep–wake cycle can significantly modulate mood [11].

Light can affect mood in several ways: by directly modulating the availability of neurotransmitters such as serotonin, which is involved in mood regulation, and by entraining and stabilising circadian rhythms, thereby addressing circadian desynchronisation and sleep disorders, which are rather common in people suffering from mental disorders. Therefore, in the last decades, light as an intervention—light therapy—has found an increasingly widespread use for treating mood and other psychiatric disorders [73, 97].

The precise mechanisms by which light exerts a positive influence on mood are currently not known though. In addition to the circadian effects of light mediated via the SCN, a pathway from the retina to the habenula has recently been found to be involved in mediating effects of light on mood in animal models [38, 110]. This pathway, connecting some ipRGCs with the habenula and bypassing the SCN altogether, has been suggested to specifically mediate light-induced alterations in mood [38]. Although it is unclear to what extent these findings can be applied to humans, imaging studies at least suggest that the human habenula is also sensitive to modulations of ambient light [46]. More research is needed to identify the mechanisms underlying light therapy.

In the following, we will provide an overview of the major clinical applications of light therapy and a brief guide to its use in daily clinical practice.

## Light therapy as an intervention in psychiatric conditions

Bright light therapy (BLT) for mood disorders was first introduced for the treatment of Seasonal Affective Disorder (SAD) in 1984 [68]. SAD is a subtype of depression characterised by strong seasonal variations in mood states. BLT is nowadays established as first-line treatment for SAD [61, 75] leading to an amelioration of symptoms after a few days of treatment. Light therapy is also effective as second-line treatment for non-seasonal depression, although it usually takes longer (2–5 weeks) than in SAD to achieve a therapeutic effect [2, 75, 87]. BLT, especially in combination with selective serotonin reuptake inhibitors (SSRIs), can accelerate the clinical improvement and lead to significantly fewer residual symptoms [7, 53]. In patients with chronic depression, BLT has been shown to lead to remarkable remission rates compared to placebo [41] and represents a valid therapeutic option also in gender-related mood disorders, such as premenstrual dysphoric disorder and perinatal depression [47, 96].

BLT can be delivered by special, commercially available therapy lamps, which operate at illuminance levels between 7000 and 10,000 lx, but natural daylight during a regular one-hour morning walk has been shown to be similarly effective [99]. In populations who suffer from depressive mood resulting from of a lack of exposure to natural daylight due to, for example, working duties in shift workers, patients with altered sleep–wake rhythms (e.g. delayed sleep–wake phase disorder), or social withdrawal (patients with psychiatric disorders, elderly people), BLT provides an effective treatment and valid alternative to pharmacological approaches [98].

Not only “active” chronotherapeutic approaches, but also an adequate architectural design of the light environment may have relevant clinical implications for psychiatric patients. The availability of light in hospital rooms has been shown to decrease the length of stay of depressed patients in a clinic [6]. Moreover, retrospective analyses revealed a three-day shorter hospitalisation in bipolar depressed inpatients exposed to natural light in sunny hospital rooms compared to those in darker rooms [8].

## Light therapy as an intervention in other medical conditions

In recent years, light therapy has been increasingly implemented as an adjunctive therapy for several other medical conditions. In patients with anorexia or bulimia nervosa, light not only improves mood but also helps to better control specific disease-related symptoms (for a review see [5, 49]). Well-controlled longitudinal studies have demonstrated that light not only has antidepressant effects in age-related depression, but can also slow down the progressive cognitive decline in dementia [52, 65]. More generally, due to its rhythm-synchronising properties and its enhancing effects on sleep quality and wakefulness, BLT is becoming an important tool in geriatric care, to treat sleep–wake disturbances and reduce general listlessness [76]. The stabilising effects of light also make BLT a useful additional treatment in adult attention deficit hyperactivity disorder (ADHD) [69], borderline personality disorder [19], and other conditions characterised by sleep–wake disruption, such as schizophrenia [18] or neurodegenerative diseases [103]. New applications are also emerging in internal medicine, e.g. in intensive care units, where day and night differences in lighting are often severely attenuated, which may result in patients developing a fragmented sleep–wake cycle with a negative impact on their recovery [36]. Studies have also demonstrated beneficial effects of BLT in patients with sleep–wake abnormalities after renal transplantation [21] or in cirrhotic patients [33], as well as in severely brain-injured patients in post-comatose states [9, 10], and Parkinson’s disease [90]. Finally, one of the most common applications of light, often in combination with exogenous melatonin, is found in sleep medicine [70, 71] for the treatment of specific circadian rhythm sleep–wake disorders (CRSWD), including advanced and delayed sleep–wake phase disorder, jet lag, shift work, sighted non-24 and irregular sleep–wake phase disorder (for diagnostic criteria see [89]).

## Light therapy in practice

### Timing, frequency and duration of light therapy sessions

The antidepressant effect of light is most pronounced when it is administered in the early morning hours [86, 97]. For CRSWD, the timing of therapeutic light exposure depends on the type of circadian disturbance and the direction of phase shift (advance or delay) to be pursued in order to achieve circadian resynchronisation. Therefore, a reliable marker of circadian phase should be first assessed to identify the phase position and then determine the timing of light treatment. The gold standard for measuring circadian phase is obtained by quantifying the so-called dim light melatonin onset (DLMO), i.e. the time at which melatonin levels rise above baseline, indicating that melatonin secretion has started. However, implementing DLMO assessment in the clinical practice remains difficult due to the limited availability of equipped centres that perform melatonin analyses and the costs of this diagnostic procedure, which are currently not reimbursed by health insurances in most European countries.

BLT is particularly effective when exposure to light occurs regularly, i.e. on a daily basis, for at least 30–60 min. Therefore, it is commonly performed in a domestic setting, which facilitates the required compliance, especially regarding timing, frequency, and duration of the treatment sessions. Disease relapses due to lacking therapeutic adherence depend on the underlying pathological condition: while SAD may rapidly reappear after a short therapy break, isolated days without light therapy are unlikely to have any negative consequences on circadian rhythm stabilisation in CRSWD, if regular sleep-wake schedules are maintained.

### Light therapy devices

Most light therapy devices on the market are suitable for clinical use. They reach a corneal illuminance of 7000–10,000 lx at a viewing distance of 20–35 cm and are equipped with a protective screen with almost complete UV filtering. Ideally designed devices illuminate the patient diagonally from above with an irradiation angle of ~ 15°. A bevelled light surface prevents annoying glare and allows simultaneous reading, thus being better tolerated. To obtain a therapeutic effect, it is not necessary to look directly into the light source, but the eyes must be open. Available light therapy glasses, which even allow mobility during the sessions, also partially meet the required criteria of sufficient light illuminance. However, most of them have not yet been evaluated in large, randomised clinical trials. Another alternative to receive light in the early morning hours is through dawn simulators. These devices start providing a relatively weak light signal about 90 min before wake-up time, which, covering the patients’ final sleep cycle, then gradually increases in intensity from about 0.001 lx to about 300 lx. However, also for these devices, the design plays an important role, as a diffuse, wide lighting area is necessary to reach the sleeper in the different lying positions. For the same reason, other types of available miniature lighting devices are not recommended because of their small luminous field [98].

### Adverse reactions

Adverse reactions to light therapy include eye irritation, blurry vision, grumpiness, headache or nausea after light exposure. However, these effects are usually rare and lessen after a few days of treatment or under reduced dosage [98]. Isolated cases of increased excitability following light therapy have been reported in patients with bipolar disorder [98]. Occurring sleep problems such as problems related to initiating sleep when light is administered in the evening, or early morning awakenings when light is administered in the morning, are mostly related to an unappropriated time of light exposure and can be quickly resolved by modifying the timing of light therapy sessions.

### Contraindications

Some relative contraindications should be taken into account when considering light therapy in patients with ophthalmological diseases or taking photosensitising drugs. These are summarised in Table 1; [22, 40].

**Table 1:** Relative contraindications to light therapy. (Modified from [22])

Ophthalmological examination recommended in the following conditions	– Pre-existing diseases of the retina or the eye, e.g. retinal detachment, retinitis pigmentosa, glaucoma – Systematic diseases affecting the retina, e.g. diabetes mellitus – Previous cataract surgery or lens removal – Elderly people (increased risk of age-related macular degeneration; AMD)
Caution needed by patients taking following photosensitizing drugs	– Neuroleptics (phenothiazines) – Antidepressants (imipramine) – Mood stabilizers (lithium) – Diuretics (hydrochlorothiazide) – 8‑methoxypsoralen – Cardiac medications (propranolol, amiodarone) – Chloroquine – Antibiotics (tetracycline) – “Natural medicines” (melatonin, St. John’s Wort)

## Summary

Light not only enables us to see fine detail, colour and motion, but also exerts non-visual effects on circadian rhythms, sleep and mood. Light at the wrong time may disrupt circadian rhythms and sleep, but in the form of light therapy, light exposure can be used as an intervention for psychiatric and other medical conditions.

## Abbreviations

ADHD: Attention deficit hyperactivity disorder
BLT: Bright light therapy
CCT: Correlated colour temperature
CIE: Commission Internationale de l’Eclairage
CRSWD: Circadian rhythm sleep-wake disorders
DLMO: Dim-light melatonin onset
EEG: Electroencephalogram
GHT: Geniculohypothalamic tract
IGL: Intergeniculate leaflet
ipRGC: Intrinsically photosensitive retinal ganglion cell
LED: Light-emitting diode
PRC: Phase response curve
RGC: Retinal ganglion cell
RHT: Retinohypothalamic tract
RN: Raphe nuclei
SAD: Seasonal affective disorder
SCN: Suprachiasmatic nuclei
SSRI: Selective serotonin reuptake inhibitor
SWA: Slow wave activity
UV: Ultraviolet
